# SARS-CoV-2 infection rewires host cell metabolism and is potentially susceptible to mTORC1 inhibition

**DOI:** 10.1038/s41467-021-22166-4

**Published:** 2021-03-25

**Authors:** Peter J. Mullen, Gustavo Garcia, Arunima Purkayastha, Nedas Matulionis, Ernst W. Schmid, Milica Momcilovic, Chandani Sen, Justin Langerman, Arunachalam Ramaiah, David B. Shackelford, Robert Damoiseaux, Samuel W. French, Kathrin Plath, Brigitte N. Gomperts, Vaithilingaraja Arumugaswami, Heather R. Christofk

**Affiliations:** 1 Department of Biological Chemistry, David Geffen School of Medicine, University of California, Los Angeles (UCLA), Los Angeles, CA USA; 2 Department of Molecular and Medical Pharmacology, UCLA, Los Angeles, CA USA; 3 UCLA Children’s Discovery and Innovation Institute, Mattel Children’s Hospital UCLA, Department of Pediatrics, David Geffen School of Medicine, UCLA, Los Angeles, CA USA; 4 Department of Pulmonary and Critical Care Medicine, David Geffen School of Medicine, UCLA, Los Angeles, CA USA; 5 Department of Ecology and Evolutionary Biology, University of California, Irvine, Irvine, CA USA; 6 Jonsson Comprehensive Cancer Center, UCLA, Los Angeles, CA USA; 7 California Nanosystems Institute, UCLA, Los Angeles, CA USA; 8 Department of Bioengineering, UCLA Samueli School of Engineering, Los Angeles, CA USA; 9 Department of Molecular Biology Interdepartmental Program, UCLA, Los Angeles, CA USA; 10 Department of Pathology and Laboratory Medicine, David Geffen School of Medicine, UCLA, Los Angeles, CA USA; 11 Eli and Edythe Broad Stem Cell Research Center, UCLA, Los Angeles, CA USA

**Keywords:** TOR signalling, Mechanisms of disease, Mucosal immunology, Viral infection

## Abstract

Viruses hijack host cell metabolism to acquire the building blocks required for replication. Understanding how SARS-CoV-2 alters host cell metabolism may lead to potential treatments for COVID-19. Here we profile metabolic changes conferred by SARS-CoV-2 infection in kidney epithelial cells and lung air-liquid interface (ALI) cultures, and show that SARS-CoV-2 infection increases glucose carbon entry into the TCA cycle via increased pyruvate carboxylase expression. SARS-CoV-2 also reduces oxidative glutamine metabolism while maintaining reductive carboxylation. Consistent with these changes, SARS-CoV-2 infection increases the activity of mTORC1 in cell lines and lung ALI cultures. Lastly, we show evidence of mTORC1 activation in COVID-19 patient lung tissue, and that mTORC1 inhibitors reduce viral replication in kidney epithelial cells and lung ALI cultures. Our results suggest that targeting mTORC1 may be a feasible treatment strategy for COVID-19 patients, although further studies are required to determine the mechanism of inhibition and potential efficacy in patients.

## Abstract (web summary)

The pandemic of COVID-19, caused by SARS-CoV-2 infection, warrants immediate investigation for therapy options. Here the authors show, using epithelial and air-liquid interface cultures, that SARS-CoV-2 hijacks host cell metabolism to facilitate viral replication, and that inhibition of mTORC1, a master metabolic regulator, suppresses viral replication.

## Introduction

Severe acute respiratory syndrome-related coronavirus 2 (SARS-CoV-2), like all viruses, requires host cells to provide the building blocks for viral replication, and viruses can achieve this by rewiring host cell metabolism^1–3^. Viruses increase and divert production of nucleotides, lipids, amino acids, and other metabolites away from host processes to build viral particles. Viruses can increase anabolism in multiple ways. For example, Zika virus increases synthesis of glucose-derived nucleotides in mosquito, but not human, cells^4^, and adenovirus increases anabolic metabolism through activation of MYC^5^.

Understanding how SARS-CoV-2 alters host cell metabolism could lead to potential treatments for coronavirus disease 2019 (COVID-19), the disease it causes^6^. Clinical data suggest that patients with underlying metabolic diseases such as type 2 diabetes are at greater risk of developing more severe symptoms than patients without underlying metabolic conditions, suggesting a role for metabolism in SARS-CoV-2 infection and COVID-19^6^.

The TCA cycle produces metabolites that are used to make amino acids, lipids, and nucleotides^7^, which viruses require for replication. mTORC1 is known to regulate anabolic metabolism and mitochondrial activity, and mTORC1 inhibitors can decrease the levels of TCA cycle metabolites^8^.

Here, we describe that SARS-CoV-2 infection rewires carbon entry into the TCA cycle, reducing oxidative glutamine metabolism and increasing pyruvate entry via pyruvate carboxylase (PC). We use this information to also show that mTORC1 activity is increased during SARS-CoV-2 infection and provide evidence that mTORC1 inhibitors decrease SARS-CoV-2 levels in multiple systems. These data offer a rationale for a potential therapeutic option for treating COVID-19 patients.

## Results

### Oxidative use of glutamine is reduced in SARS-CoV-2 infection

To analyze the metabolic changes induced by SARS-CoV-2 infection, we first performed LC-MS based metabolomics on SARS-CoV-2-infected Vero kidney epithelial cells 24 h post infection. The cells were labeled with U-^13^C-glucose or U-^13^C-glutamine to determine if infected cells had altered carbon use into pathways that provide the metabolites needed to produce viral particles.

**Fig. 1:**
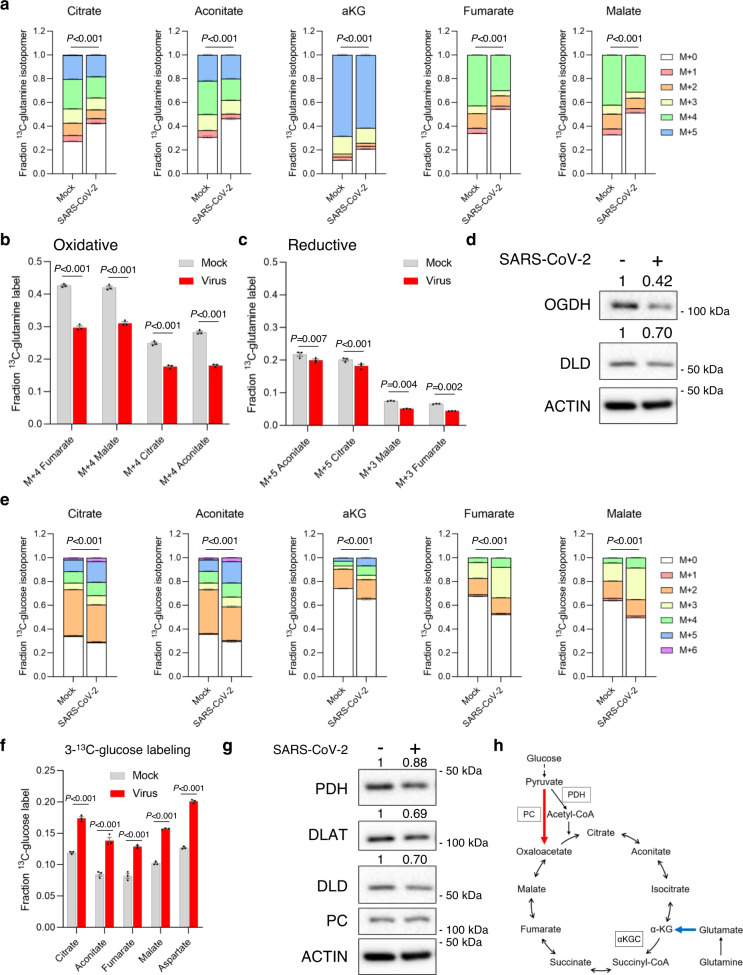
SARS-CoV-2 infection alters TCA cycle metabolism. **a** Glutamine entry into the TCA cycle is reduced in SARS-CoV-2 infected cells. Mass isotopomer analysis of TCA cycle metabolites in mock or SARS-CoV-2 infected Vero kidney epithelial cells after 24 h incubation with U-^13^C-glutamine. Each isotopomer is a different color, which is defined in the figure. *P* values compare the total fraction labeled between mock and SARS-CoV-2 infected cells (*n* = 3 biologically independent samples). **b** The oxidative TCA cycle is decreased in SARS-CoV-2 infected (red bars) Vero cells after 24 h infection compared with mock infected (gray bars) Vero cells (*n* = 3 biologically independent samples). **c** The reductive TCA cycle is maintained in SARS-CoV-2 infected (red bars) Vero cells compared with mock infected (gray bars) Vero cells after 24 h infection (*n* = 3 biologically independent samples). **d** Levels of αKGC members OGDH and DLD are reduced in SARS-CoV-2 infected Vero cells (representative of two biological independent samples). **e** Glucose-derived carbon entry into the TCA cycle is increased in SARS-CoV-2 infected cells. Mass isotopomer analysis of TCA cycle metabolites in mock or SARS-CoV-2 infected Vero kidney epithelial cells after 24 h incubation with U-^13^C-glucose. Each isotopomer is a different color, which is defined in the figure. *P* values compare the total fraction labeled between mock and SARS-CoV-2 infected cells (*n* = 3 biologically independent samples). **f** Glucose-derived carbon entry into the TCA cycle using PC is increased in SARS-CoV-2 infected cells. Fraction of labeled metabolites in mock (gray bars) or SARS-CoV-2 infected (red bars) Vero cells after 24 h incubation with 3-^13^C-glucose (*n* = 3 biologically independent samples). **g** Levels of PDH complex members are decreased in SARS-CoV-2 infected Vero cells (representative of two biological independent samples). **h** Schematic of the changes in TCA cycle metabolism in SARS-CoV-2 infected cells. Glutamine entry is reduced (blue arrow) and pyruvate entry using PC is increased (red arrow). Unless indicated, data are the mean ± s.e.m. and *P* values were obtained by two-way ANOVA with Sidak’s multiple comparison test.

Both glucose and glutamine provide carbons to the mitochondrial TCA cycle (Supplementary Fig. 1a). The TCA cycle provides metabolites for proteins, lipids, and nucleotides, and also metabolites that control posttranslational modifications of histones and other proteins^7^. It is therefore a candidate for dysregulation by SARS-CoV-2. All five detectable TCA cycle metabolites had decreased labeling of carbons derived from U-^13^C-glutamine in SARS-CoV-2 infected Vero cells (Fig. 1a). Importantly, we saw no differences in cell number between mock and SARS-CoV-2 infected cells at the time of metabolite extraction 24 h post SARS-CoV-2 infection, suggesting no measurable difference in cell death (Supplementary Fig. 1b, c). As the TCA cycle can be either oxidative or reductive, depending on the metabolic needs of the cell^9^, we asked whether SARS-CoV-2 infection altered either of these processes. The oxidative TCA cycle can use glutamine to provide four carbons to fumarate, malate, citrate, and aconitate (Supplementary Fig. 1d), and we saw decreases in these markers of the oxidative TCA cycle in SARS-CoV-2 infected cells (Fig. 1b). In contrast, the reductive TCA cycle runs in reverse and generates a different U-^13^C-glutamine labeling pattern (Supplementary Fig. 1e). SARS-CoV-2 infection only mildly reduced reductive carboxylation (Fig. 1c), suggesting that the decrease in glutamine labeling into the TCA was mostly via decreased oxidative metabolism (Fig. 1b). This is further highlighted by the labeling pattern of citrate in SARS-CoV-2 infected cells (Supplementary Fig. 1f). In addition, we confirmed the changes in U-^13^C-glutamine labeling at an earlier timepoint, 14 h post SARS-CoV-2 infection in Vero cells, where we saw decreases in markers of the oxidative TCA cycle (Supplementary Fig. 1g) and no change in reductive carboxylation (Supplementary Fig. 1h). We also confirmed the changes in glutamine metabolism in SARS-CoV-2 infected HEK293T cells overexpressing the ACE2 receptor (HEK293T-ACE2): TCA cycle metabolites had decreased labeling of carbons derived from U-^13^C-glutamine in SARS-CoV-2 infected HEK293T-ACE2 cells (Supplementary Fig. 1i). Consistent with the Vero cell data, the decrease in glutamine labeling in SARS-CoV-2 infected HEK293T-ACE2 cells was caused by a reduction in the oxidative TCA cycle (Supplementary Fig. 1j). The reductive arm of the TCA cycle was slightly increased in SARS-CoV-2 infected HEK293T-ACE2 cells (Supplementary Fig. 1k).

The labeling pattern of citrate from U-^13^C-glutamine in SARS-CoV-2 infected Vero cells (Supplementary Fig. 1f) was similar to that seen in previous work in *OGDH* knockdown cells^10^. OGDH is a component of the α-ketoglutarate (αKG) dehydrogenase complex (αKGC) that oxidizes αKG to succinyl-CoA^9^ (Supplementary Fig. 1a). To determine whether αKGC components are reduced during SARS-CoV-2 infection, we immunoblotted for OGDH and another αKGC member DLD. Both OGDH and DLD were reduced in SARS-CoV-2 infected Vero cells (Fig. 1d). We also used a previously published RNA-seq dataset from a cardiomyocyte model of SARS-CoV-2 infection^11^. We saw decreased expression of the αKGC complex members *OGDHL* and *DLD* in the SARS-CoV-2 infected cardiomyocytes, which suggests the oxidative TCA cycle is also impaired in these cells (Supplementary Fig. 1l). Reduced expression of αKGC complex members could explain the decrease in glutamine incorporation via the oxidative TCA cycle (Fig. 1b).

### Pyruvate entry using PC is increased during infection

We then asked whether changes in incorporation of glucose-derived carbons into the TCA cycle occurred in SARS-CoV-2 infected cells. We observed that SARS-CoV-2 infected Vero cells had increased U-^13^C-glucose incorporation into the TCA cycle (Fig. 1e). Glucose-derived carbons predominantly enter the TCA cycle by pyruvate conversion to acetyl-CoA using pyruvate dehydrogenase (PDH) or pyruvate conversion to oxaloacetate using PC (Supplementary Fig. 2a)^12^. We saw an increase in M+3 and M+5 labeling of citrate in SARS-CoV-2 infected cells, indicating an increase of glucose carbon entry using PC (Supplementary Fig. 2b). The increase in M+3 labeling of malate in SARS-CoV-2 infected cells also suggested increased entry of carbons using PC (Supplementary Fig. 2c). We next asked if the changes in glucose carbon entry into the TCA cycle are seen in other cell systems infected with SARS-CoV-2. Using HEK293T-ACE2 cells, we confirmed that SARS-CoV-2 infection increases M+3 labeling of TCA cycle metabolites (Supplementary Fig. 2d), and that SARS-CoV-2 infection decreases M+2 labeling of citrate and aconitate (Supplementary Fig. 2e).

We then examined whether these SARS-CoV-2-induced changes in TCA cycle glucose entry occur in primary human airway derived air–liquid interface (ALI) cultures, as ALI cultures provide a more representative model of SARS-CoV-2 infection of the respiratory tract than standard in vitro cultures^13^. We generated ALI cultures from primary normal human bronchial epithelial (NHBE) cells and confirmed differentiation using markers of ciliated cells, mucus cells, and airway basal stem cells (ABSCs) (Supplementary Fig. 2f). We infected the ALI cultures with SARS-CoV-2 for 24 h, labeled with U-^13^C-glucose and saw an increase in glucose labeling of TCA cycle metabolites in SARS-CoV-2 infected cultures (Supplementary Fig. 2g). SARS-CoV-2 infected ALI cultures also exhibited increased M+3 labeling of TCA cycle metabolites (Supplementary Fig. 2h), similar to results obtained from the SARS-CoV-2 infected Vero cells and HEK293T-ACE2 cells. In addition, we confirmed the changes in U-^13^C-glucose labeling at an earlier timepoint, 14 h post SARS-CoV-2 infection in Vero cells, where we saw decreased M+3 labeling of TCA cycle metabolites (Supplementary Fig. 2i)

The labeling patterns of glucose carbon entry into the TCA cycle in multiple systems is consistent with previous work showing increased use of PC to provide carbons to the TCA cycle in cells that have reduced oxidation of glutamine in the TCA cycle^10^. We therefore used 3-^13^C-glucose labeling to confirm whether SARS-CoV-2 infection increases glucose carbon entry using PC. The labeled carbon in 3-^13^C-glucose can only enter the TCA cycle using PC and not PDH. We saw increased labeling of TCA cycle metabolites in SARS-CoV-2 infected Vero cells labeled with 3-^13^C-glucose, confirming the increased entry of carbons into the TCA using PC (Fig. 1f). To determine whether SARS-CoV-2 infection alters expression of PDH or PC, we next immunoblotted for PDH complex members and PC, and observed reduced levels of PDH complex members PDH, DLAT, and DLD in SARS-CoV-2 infected Vero cells (Fig. 1g). PC protein levels were unchanged in SARS-CoV-2 infected Vero cells. We then turned to the RNA-seq dataset of SARS-CoV-2 infected cardiomyocytes, and saw increased PC expression and decreased *PDHA1, PDHB*, and *DLD* expression in SARS-CoV-2 infected cardiomyocytes (Supplementary Fig. 1l)^11^. We also saw an increase in PC expression (Supplementary Fig. 2j) and decrease in *PDHA1* expression (Supplementary Fig. 2k) in our previously performed single-cell RNA-seq (scRNA-seq) on ALI mucociliary cells infected with SARS-CoV-2^13^. We immunoblotted protein extracts from ALI cultures and confirmed the increase in PC protein levels in SARS-CoV-2 infected cells, with no change in PDH protein expression (Supplementary Fig. 2l). These findings are consistent with our metabolomics data showing increased entry of glucose carbons into the TCA cycle using PC. Taken together, these findings suggest that SARS-CoV-2 infection upregulates PC activity to increase pyruvate entry into the TCA cycle, although further studies will be required to confirm whether PC is required for SARS-CoV-2 replication (Fig. 1h). This suggests that directly targeting PC or inhibiting pyruvate entry into the mitochondria could be a therapeutic strategy against COVID-19, however there are currently no approved drugs that target PC or the mitochondrial pyruvate carrier.

### mTORC1 activity is increased during SARS-CoV-2 infection

The urgent need for antiviral therapies toward SARS-CoV-2 suggests that benefits could be provided by repurposing drugs that are already approved for other indications. mTORC1 is a master regulator of anabolic metabolism that is targeted by three FDA-approved drugs: rapamycin, everolimus, and temsirolimus^14^. Multiple viruses can increase mTORC1 activity to increase the synthesis of materials required for viral replication^15,16^. Recent studies also show SARS-CoV-2 increases activation of kinases upstream of mTORC1^17^, and that SARS-CoV-2 proteins can bind to mTORC1 regulators^18^. mTORC1 is therefore a strong candidate to target as a COVID-19 therapeutic.

**Fig. 2:**
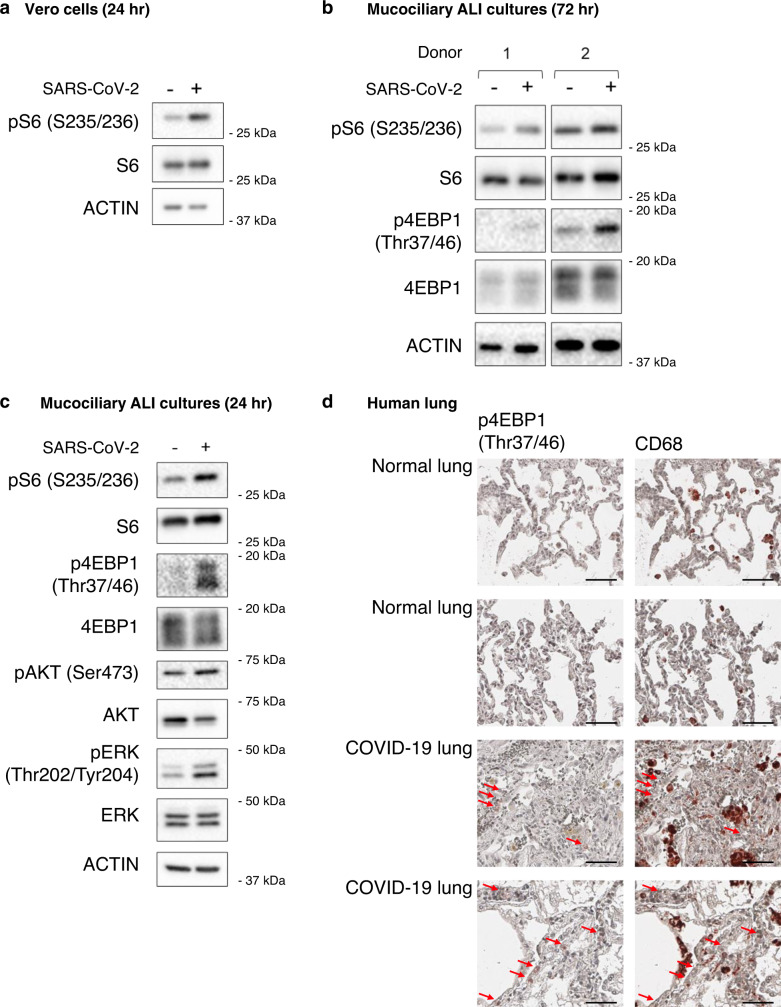
SARS-CoV-2 infection increases mTORC1 activity. **a** mTORC1 activity is increased in SARS-CoV-2 infected Vero kidney epithelial cells. Protein was extracted from Vero cells infected with SARS-CoV-2 for 24 h and immunoblotted for the indicated proteins (representative of two biological independent samples). **b** mTORC1 activity is increased in ALI mucociliary cultures derived from two different donors. Protein was extracted from ALI cultures infected with SARS-CoV-2 for 72 h and immunoblotted for the indicated proteins (*n* = 2 biological independent samples). **c** pAKT and pERK are increased in SARS-CoV-2 infected NHBE ALI cultures (representative of two biological independent samples). **d** mTORC1 activity is increased in COVID-19 patient lungs. IHC staining of p4EBP1 and the macrophage marker CD68 on lungs from COVID-19 and non-COVID-19 patients. Images are representative of two out of five COVID-19 patient lungs and nine out of nine non-COVID-19 lungs. Red arrows indicate cells positive for p4EBP1. Scale bar 50 µM.

We first tested whether SARS-CoV-2 infection increases mTORC1 activity in Vero kidney epithelial cells. SARS-CoV-2 infected cells showed an increase in pS6 levels 24 h after infection, indicating an increase in mTORC1 activity (Fig. 2a). We also confirmed that mTORC1 activity began to increase by 16 h post infection, a timepoint when we first detected expression of SARS-CoV-2 proteins (Supplementary Fig. 3a). We then tested whether SARS-CoV-2 could increase mTORC1 activity in our primary human ABSC derived ALI cultures. We generated ALI cultures from two different donors and infected them with SARS-CoV-2 for 3 days, a timepoint early in infection when there is minimal cell death. We saw similar mean infection rate to our previously published work, although we had a smaller range of infectivity, which is expected as the cultures were derived from different donors (Supplementary Fig. 3b)^13^. The lower infectivity of ALI cultures is a strength of this model as it is more reflective of what occurs in patients, where only a small percentage of patient lung cells are infected. Both ALI cultures showed increased levels of pS6 after SARS-CoV-2 infection and an increase in levels of p4EBP1 (Fig. 2b). We also generated ALI cultures from primary NHBE cells, and again observed increased levels of pS6 and p4EBP1 after 24 h SARS-CoV-2 infection (Fig. 2c). To better understand how mTORC1 is getting activated in SARS-CoV-2 infected cells, we examined activation of kinases upstream of mTORC1. We found increased pERK and pAKT levels in SARS-CoV-2 infected ALI cultures (Fig. 2c), consistent with SARS-CoV-2 activation of signaling pathways upstream of mTORC1, although more work is required to fully elucidate the mechanism and whether there are contributions by cytokine release or infection-independent effects. These data show that SARS-CoV-2 infection increases mTORC1 activity in vitro, and the increased pERK and pAKT levels detected complement recent work that showed PI3K activity is increased in SARS-CoV-2 infected cells^17^.

Next, we sought to determine whether mTORC1 activity could be increased in the lungs of COVID-19 patients. We obtained lung samples from COVID-19 patient autopsies and control lungs from non-COVID-19 patient autopsies. Evidence for p4EBP1 could only be found in COVID-19 autopsies (2/5) and not in non-COVID-19 lungs (0/9) (Fig. 2d). These findings suggest that mTORC1 activity can be increased in the lungs of COVID-19 patients. Although it would be useful to determine mTORC1 activity in lung biopsies from living COVID-19 patients, it is not possible to obtain lung tissue from these patients. Since macrophages have been shown to be present in the lungs of COVID-19 patients^19^, we stained for CD68, a marker of macrophages, to determine whether the p4EBP1 positive cells in the COVID-19 lung biopsies are macrophages. Although we did see increased CD68 levels in the COVID-19 lungs, we did not see evidence of p4EBP1 staining in CD68 positive cells (Fig. 2d), suggesting that the cells with mTORC1 activation in the COVID-19 patient lung biopsies are not macrophages.

Since viruses can increase mTORC1 activity in multiple ways, we next wanted to confirm that the SARS-CoV-2 induced increase in mTORC1 activity could be inhibited by FDA-approved mTORC1 inhibitors. We infected Vero kidney epithelial cells with SARS-CoV-2 and saw the expected increase in mTORC1 activity (Supplementary Fig. 3c). Importantly, three different mTORC1 inhibitors prevented SARS-CoV-2 from activating mTORC1 (Supplementary Fig. 3c), suggesting that SARS-CoV-2 may be susceptible to mTORC1 inhibitors as antivirals.

### mTORC1 inhibitors reduce SARS-CoV-2 levels in ALI cultures

**Fig. 3:**
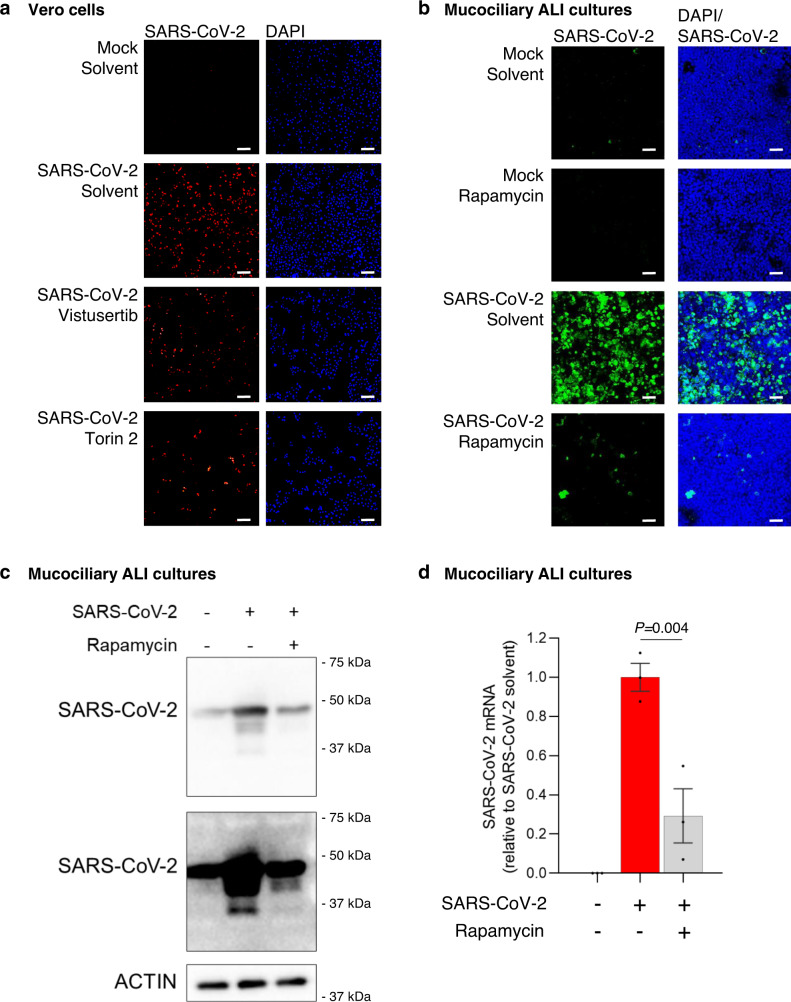
mTORC1 inhibitors reduce SARS-CoV-2 replication. **a** mTORC1 inhibitors reduce SARS-CoV-2 levels in Vero kidney epithelial cells. Immunofluorescence of cells infected with SARS-CoV-2 and treated with 25 nM of the indicated non-FDA approved mTORC1 inhibitors for 72 h. Cells were stained for dsRNA (red) to show SARS-CoV-2 levels. DAPI (blue) stained the nuclei of the Vero cells (*n* = 3 biologically independent samples). **b** The FDA-approved mTORC1 inhibitor rapamycin reduces SARS-CoV-2 levels in NHBE ALI mucociliary cultures. Immunofluorescence of cells infected with SARS-CoV-2 and treated with 1 µM rapamycin for 48 h. Cells were stained with an anti-SARS coronavirus antibody (green) to show SARS-CoV-2 levels. DAPI (blue) stained the nuclei of the mucociliary cells (*n* = 3 biologically independent samples). **c** FDA-approved mTORC1 inhibitors reduce SARS-CoV-2 protein expression. NHBE ALI cultures were infected with SARS-CoV-2 and treated with 1 µM rapamycin. Protein was extracted after 48 h and immunoblotted with an anti-SARS coronavirus antibody to determine SARS-CoV-2 protein expression. Multiple exposures of SARS-CoV-2 antibody are shown (representative of three biological independent samples). **d** FDA-approved mTORC1 inhibitors reduce SARS-CoV-2 gene expression. NHBE ALI cultures were infected with SARS-CoV-2 (red bar) and treated with 1 µM rapamycin (gray bar). RNA was extracted after 48 h and SARS-CoV-2 *N* gene expression quantified relative to *RPLP0* using qPCR. Data are normalized to SARS-CoV-2 solvent cells (*n* = 3 biologically independent samples. Data are mean ± s.e.m. and *P* values were obtained by one-way ANOVA with Tukey’s multiple comparison’s test). Scale bar 30 µM.

After confirming the on target effect of mTORC1 inhibitors in SARS-CoV-2 infected Vero kidney epithelial cells, we next asked whether mTORC1 inhibitors could reduce SARS-CoV-2 replication. We first treated mock or SARS-CoV-2 infected Vero kidney epithelial cells with the mTORC inhibitors vistusertib or torin 2. We stained cells for dsRNA, a marker of the SARS-CoV-2 RNA genome, and saw that both vistusertib and torin 2 reduced SARS-CoV-2 replication in infected cells (Fig. 3a). Vistusertib and torin 2 are not approved for use in any indication in humans, so we next determined whether FDA-approved mTORC1 inhibitors could also reduce SARS-CoV-2 replication. We first confirmed that rapamycin, everolimus, and temsirolimus reduced SARS-CoV-2 levels in Vero kidney epithelial cells by measuring the expression of the SARS-CoV-2 *N* gene. All three inhibitors reduced SARS-CoV-2 *N* gene expression in infected cells (Supplementary Fig. 4a). We also observed mildly decreased expression of SARS-CoV-2 protein levels in mTORC1 inhibitor treated Vero cells (Supplementary Fig. 4b). We then saw that SARS-CoV-2 levels were decreased in HEK293-ACE2 cells treated with the FDA-approved mTORC1 inhibitors rapamycin, everolimus, and temsirolimus (Supplementary Fig. 4c). Taken together, these data suggest that FDA-approved mTORC1 inhibitors have antiviral effects on SARS-CoV-2.

We next used our ALI cultures to examine whether FDA-approved mTORC1 inhibitors have antiviral effects toward SARS-CoV-2 in a more representative model of patient SARS-CoV-2 infection. We used rapamycin as it caused the largest reduction in SARS-CoV-2 levels in infected Vero kidney epithelial cells, and we observed a large reduction in SARS-CoV-2 levels in ALI cultures treated with rapamycin (Fig. 3b). We also saw that rapamycin treatment reduced SARS-CoV-2 levels when it was added 2 h after SARS-CoV-2 infection (Supplementary Fig. 4d). These data suggest that rapamycin can inhibit the synthesis of new viral components, but it is possible that rapamycin may also be able to inhibit viral particle uptake. Finally, rapamycin treatment reduced expression of SARS-CoV-2 protein levels in ALI cultures (Fig. 3c) and reduced expression of the SARS-CoV-2 *N* gene (Fig. 3d). These results collectively demonstrate that FDA-approved mTORC1 inhibitors may be useful as antivirals during the treatment of COVID-19.

## Discussion

The hijacking of host cell metabolism by SARS-CoV-2 offers opportunities to target pathways essential to the virus life cycle. We show that oxidative TCA metabolism of glutamine is decreased in SARS-CoV-2 infected cells, and glucose-derived carbon entry into the TCA cycle via PC is increased in SARS-CoV-2 infected cells, although additional work is required to determine the destination of these carbons and whether these changes in TCA metabolism contribute to and are necessary for SARS-CoV-2 replication. It is possible that SARS-CoV-2 increases PC activity to maintain synthesis of aspartate from oxaloacetate even when the oxidative TCA cycle is decreased. Maintaining aspartate synthesis would be advantageous to SARS-CoV-2 as it is used to synthesize nucleotides^20,21^, which SARS-CoV-2 requires to replicate its genome. Aspartate is also used to generate asparagine, which is necessary to maintain mTORC1 activity^22^, and both aspartate and asparagine are required to provide amino acids for viral mRNA translation. Therefore, targeting aspartate synthesis might offer a therapeutic strategy against COVID-19.

SARS-CoV-2 infected cells had increased labeling of citrate by glucose-derived carbons, but also maintained citrate labeling by reductive carboxylation. This may enable citrate to be used for fatty acid synthesis, a process that occurs in coronaviruses and other viruses^23,24^. It would be informative to perform lipidomic studies investigating the changes in lipid synthesis during SARS-CoV-2 infection in future studies.

Increased mTORC1 activity is a hallmark of increased anabolic metabolism, and this study shows that SARS-CoV-2 infection increases mTORC1 activity. It is not known how SARS-CoV-2 increases mTORC1 activity, but recent work shows increased activation of PI3K in SARS-CoV-2 infected cells^17^, which could cause an increase in mTORC1 signaling. We also show that SARS-CoV-2 replication is reduced by FDA-approved mTORC1 inhibitors, although further work is required to determine: (1) how mTORC1 inhibitors reduce viral replication; (2) whether this strategy will be efficacious in COVID-19 patients; and (3) which cohort of COVID-19 patients may benefit most from this approach. Severe cases of COVID-19 are characterized by an overactive immune response and a cytokine storm^25^. As mTORC1 inhibitors can dampen the immune response, it is possible that they may have the most efficacy in severe COVID-19 patients when they can act as both antivirals and immunosuppressants.

## Methods

### Cell lines and culture conditions

Vero (ATCC CRL1586) and HEK293-ACE2 cells (generated from HEK293T cells from ATCC CRL3216) were cultured in DMEM supplemented with 10% FBS and 1% penicillin–streptomycin. Both cell lines were cultured at 37 °C and 5% CO_2_.

### Human tissue procurement

Large airways and bronchial tissues were acquired from deidentified normal human donors after lung transplantations at the Ronald Reagan UCLA Medical Center. Tissues were procured under Institutional Review Board-approved protocols at the David Geffen School of Medicine at UCLA, and received written and informed consent from the patients or relatives. For the rapamycin experiment NHBE cells were obtained from Lonza (CC-2540) and all samples were deidentified.

### NHBE cell culture

We seeded 85,000 NHBEs onto collagen coated transwells and grew them in the submerged phase of culture for 4–5 days in PneumaCult Ex Plus media (Stem Cell Technologies) with 500 μL media in the basal chamber and 200 μL media in the apical chamber. ALI cultures were then maintained for 21 days with only 500 μL PneumaCult ALI media (Stem Cell Technologies) in the basal chamber, and media changed every 2 days. Cultures were maintained at 37 °C and 5% CO_2_.

### ABSCs isolation

Large airways and bronchial tissues were acquired from deidentified normal human donors after lung transplantations at the Ronald Reagan UCLA Medical Center. Tissues were procured under Institutional Review Board-approved protocols at the David Geffen School of Medicine at UCLA, and all patients or relatives gave written and informed consent to the donation. Human ABSCs were isolated following a previously published method by our laboratory^26^. We incubated airways in 16 U mL^−1^ dispase for 30 min at room temperature and then 0.5 mg mL^−1^ DNase for 30 min at room temperature. Then, we stripped the epithelium and generated a single-cell suspension by incubating for 30 min in 0.1% Trypsin-EDTA at 37 °C with shaking. Isolated cells were passed through a 40 μm strainer and plated for ALI cultures.

### ALI cultures

Twenty four-well 6.5 mm transwells with 0.4 μm pore polyester membrane inserts were coated with 0.2 mg/mL collagen type I dissolved in 60% ethanol and allowed to air dry. We seeded 100,000 ABSCs onto collagen coated transwells and grew them in the submerged phase of culture for 4–5 days with 500 μL media in the basal chamber and 200 μL media in the apical chamber. ALI cultures were then cultured with only 500 μL media in the basal chamber, and media changed every 2 days. ALI cultures were maintained at 37 °C and 5% CO_2_.

### Tracheal epithelial cell (TEC) plus and serum-free media

Human ABSCs were grown in TEC Plus media and TEC serum-free media during the submerged and ALI phases of culture, respectively. TEC base media are DMEM/Ham’s F12 50/50 (Corning 15090CV).

### SARS-CoV-2 infection

All experiments used SARS-CoV-2 isolate USA-WA1/2020 (Biodefense and Emerging Infectious Resources of National Institute of Allergy and Infectious Diseases). We passaged SARS-CoV-2 in Vero cells and stored aliquots at −80 °C. We measured virus titer in Vero cells using a TCID50 assay. All SARS-CoV-2 infections were performed in the UCLA BSL3 high-containment facility with appropriate institutional biosafety approvals. Vero cells and ALI cultures were infected with SARS-CoV-2 MOI of 1 for metabolomics experiments and 0.1 for all other experiments, and media alone were used for mock infections. After 2 h, the virus containing media were removed and replaced with fresh media.

### mTORC1 inhibitor treatments

Unless stated, we pretreated Vero, HEK293-ACE2, and ALI cultures for 1 h with the indicated mTORC1 inhibitors or vehicle control. We then infected with SARS-CoV-2 for 2 h, removed the virus, and added back the inhibitors. Rapamycin (LC Laboratories R-5000), everolimus (Sigma Aldrich sml2282), and temsirolimus (Sigma Aldrich pz0020) were used at 1 and 5 µM, and torin 2 (Sigma Aldrich sml1224) and vistusertib (Selleckchem s2783) were used at 25 nM. RNA and protein were harvested after 24 h treatment, and cells were fixed for immunocytochemistry after 72 h treatment.

### Intracellular metabolite extraction

Vero cells were seeded in 12-well plates in normal media. Cells were infected with SARS-CoV-2 for 2 h, at which point the virus was removed and the media replaced with DMEM containing 10 mM U-^13^C-glucose (Cambridge Isotopes), 10 mM 3-^13^C-glucose, or 4 mM U-^13^C-glutamine (Cambridge Isotopes). ALI culture media were serum free, and Vero and HEK293T-ACE2 media contained 10% dialyzed FBS. To extract metabolites, we washed the cells with ice-cold 150 mM ammonium acetate, pH 7.3, and then added 500 μL 80% methanol and incubated for 20 min at −80 °C. Cells were then scraped off the plate, vortexed, and centrifuged for 10 min at maximum speed. We dried 400 μL of the supernatant under vacuum and stored the dried metabolites at −80 °C.

### Mass spectrometry-based metabolomics analysis

We reconstituted dried metabolites in 100 µL of a 50% acetonitrile (ACN) 50% dH_2_O solution, and then vortexed and spun them down for 10 min at maximum speed. We then transferred 70 µL of the supernatant to HPLC glass vials and injected 10 µL of these metabolite solutions per analysis. We ran the samples on a Vanquish (Thermo Scientific) UHPLC system using mobile phase A (20 mM ammonium carbonate, pH 9.7) and mobile phase B (100% ACN) at a flow rate of 150 µL/min on a SeQuant ZIC-pHILIC Polymeric column (2.1 × 150 mm, 5 μm, EMD Millipore) at 35 °C. Separation was achieved using a linear gradient from 20% A to 80% A in 20 min followed by a linear gradient from 80% A to 20% A from 20 to 20.5 min. 20% A was then held from 20.5 to 28 min. The UHPLC was coupled to a Q Exactive (Thermo Scientific) mass analyzer running in polarity switching mode with spray-voltage = 3.2 kV, sheath-gas = 40, aux-gas = 15, sweep-gas = 1, aux-gas-temp = 350 °C, and capillary-temp = 275 °C. Mass scan settings for both polarities were kept at full-scan-range = 70–1000, ms1-resolution = 70,000, max-injection-time = 250 ms, and AGC-target = 1E6. Each of the resulting “.RAW” files was then centroided and converted into two “.mzXML” files (one for positive scans and one for negative scans) using msconvert from ProteoWizard^27^. We then imported the “.mzXML” files MZmine 2 software package^28^. Ion chromatograms were generated from MS1 spectra via the built-in automated data analysis pipeline (ADAP)^29^ chromatogram module and peaks were detected via the ADAP wavelets algorithm. Peaks were aligned across all samples via the random sample consensus aligner module, gap-filled, and assigned identities using an exact mass MS1(+/−15ppm) and retention time RT (+/−0.5 min) search of our in-house MS1-RT database. Peak boundaries and identifications were then further refined by manual curation. Peaks were quantified by area under the curve integration and exported as CSV files. For stable isotope tracing, the peak areas were additionally processed via the R package AccuCor^30^ to correct for natural isotope abundance. We normalized peak areas for each sample by the measured area of an internal standard, trifluoromethanesulfonate, included in the extraction buffer and by the number of cells present in the extracted well.

### Cell lysis and immunoblotting

We lysed cells with buffer containing 50 mM Tris pH 7.4, 1% Nonidet P-40, 0.25% sodium deoxycholate, 1 mM EDTA, 150 mM NaCl, 1 mM dithiothreitol, 1 mM sodium orthovanadate, 20 mM sodium fluoride, 10 mM beta-glycerophosphate, 10 mM sodium pyrophosphate, 2 μg mL^−1^ aprotinin, 2 μg mL^−1^ leupeptin, and 0.7 μg mL^−1^ pepstatin. We performed western blot analysis using standard protocols, and probed using the following commercial antibodies: phospho-S235/236 S6 ribosomal protein (Cell Signaling Technology 4858, 1:3000), S6 ribosomal protein (Cell Signaling Technology 2217, 1:1000), 4EBP1 (Cell Signaling Technology 9644, 1:500), phospho-Thr37/46 4EBP1 (Cell Signaling Technology 2855, 1:500), AKT (Cell Signaling Technology 4691, 1:1000), phospho-Ser473 AKT (Cell Signaling Technology 9271, 1:1000), ERK (Cell Signaling Technology 9102, 1:1000), phospho-Thr202/Tyr204 ERK (Cell Signaling Technology 4370, 1:1000), OGDH (Cell Signaling Technology 26865, 1:1000), DLD (Thermo Fisher PA5-70397, 1:1000), DLAT (Cell Signaling Technology 12362, 1:1000), PC (Protein Tech 16588-1-AP, 1:10,000), PDH (Cell Signaling Technology 3205, 1:1000), SARS-CoV antibody (BEI Resources, NIAID, NIH, NR-10361, 1:10,000), and β-ACTIN (Cell Signaling Technology 3700, 1:1000). Antibody details are given in Supplementary Table 1.

### Immunocytochemistry

We determined SARS-CoV-2 levels at 72 h post infection using SARS-CoV antibody (BEI Resources, NIAID, NIH, NR-10361, 1:400), anti-SARS-CoV S Protein (similar to 240C) (BEI Resources, NIAID, NIH, NR-616, 1:100), and anti-dsRNA (Absolute Antibody, Ab01299-2.0, 1:100). We fixed Vero, HEK293-ACE2, and ALI cells with 4% paraformaldehyde for 15 min and permeabilized with 0.5% Triton X for 10 min. We then blocked for 1 h at room temperature with serum-free protein block (Dako X090930) and incubated with primary antibody overnight. Secondary antibodies were incubated for 1 h, and slides were mounted using Vectashield hardest mounting medium with DAPI (Vector Labs H-1500). We used LSM700 and LSM880 Zeiss confocal microscopes to obtain images. Antibody details are given in Supplementary Table 1.

### Immunohistochemistry

Fixed autopsy lungs were processed and embedded by TPCL at UCLA. We stained human lungs using antibodies (Supplementary Table 1) to phospho-Thr37/46 4EBP1 (Cell Signaling Technology 2855, 1:800) and CD68 (Cell Signaling Technology 76437, 1:500). Slides were scanned onto a ScanScope AT (Aperio Technologies, Inc., Vista, CA) and analyzed using QuPath software. Tissues were procured under Institutional Review Board-approved protocols at the David Geffen School of Medicine at UCLA, and consent was informed and in writing by patients or authorized relatives.

### Quantitative real-time PCR

We isolated total RNA using the Qiagen RNeasy Kit and synthesized cDNA from 700 ng RNA using the iScript Kit (Bio-Rad). We diluted cDNA to a final volume of 100 μL with ultrapure water. qPCR reactions used 2 μL cDNA, 0.5 μM primers, and Power SYBR master mix (Applied Biosystems), and were amplified on a QuantStudio5 (Applied Biosystems). We quantified the relative expression of the SARS-CoV-2 *N* gene using the ΔΔCt method with *RPLP0* as the reference gene. Primer sequences are given below and in Supplementary Table 2

*RPLP0* forward primer (5′-3′) TCTACAACCCTGAAGTGCTTGAT

*RPLP0* reverse primer (5′-3′) CAATCTGCAGACAGACACTGG

SARS-CoV-2 *N* gene forward primer (5′-3′) GACCCCAAAATCAGCGAAAT

SARS-CoV-2 *N* gene reverse primer (5′-3′) TCTGGTTACTGCCAGTTGAATCTG.

### scRNA-seq dataset analysis

We used our previously analyzed scRNA-seq of SARS-CoV-2 infected ALI cultures^13^. Gene expression was compared between infected and noninfected ALI cells in R and by the Welch two sample *t*-test.

### RNA-seq dataset analysis

We used our previously published RNA-seq gene expression data of SARS-CoV-2 infected cardiomyocytes (accession number GSE150392)^11^.

### Statistical analyses

We used two-way ANOVA with Sidak’s multiple comparison test for comparisons of isotopomers between mock and infected cells. One-way ANOVA with Tukey’s multiple comparison test was used to analyze qPCR data. *P* values < 0.05 were considered significant for all tests. Immunoblots are representative of at least two biologically independent experiments.

### Reporting summary

Further information on research design is available in the Nature Research Reporting Summary linked to this article.

## Supplementary information

Supplementary Information

Reporting Summary

## Data Availability

The data supporting the findings of this study are available from the corresponding author (H.R.C.) upon reasonable request. Source data are provided with this paper. Mass spectrometry data are available at the NIH Common Fund’s National Metabolomics Data Repository website, the Metabolomics Workbench, https://www.metabolomicsworkbench.org, where it has been assigned Project ID PR001094. The data can be accessed directly via its Project, 10.21228/M89394. The original data for Supplementary Fig. 1l are published in ref. ^11^ and are deposited at https://www.ncbi.nlm.nih.gov/geo/query/acc.cgi?acc=GSE150392. The original data for Supplementary Fig. 2j, k are published in ref. ^13^ and are deposited at https://www.ncbi.nlm.nih.gov/geo/query/acc.cgi?acc=GSE161089. Source data are provided with this paper.

## Source data

Source Data

## References

[1] Thaker SK, Ch’ng J, Christofk HR (2019). Viral highjacking of cellular metabolism. BMC Biol..

[2] DeVito SR, Ortiz-Riaño E, Martínez-Sobrido L, Munger J (2014). Cytomegalovirus-mediated activation of pyrimidine biosynthesis drives UDP-sugar synthesis to support viral protein glycosylation. Proc. Natl Acad. Sci. USA.

[3] Vastag L, Koyuncu E, Grady SL, Shenk TE, Rabinowitz JD (2011). Divergent effects of human cytomegalovirus and herpes simplex virus-1 on cellular metabolism. PLoS Pathog..

[4] Thaker SK (2019). Differential metabolic reprogramming by Zika virus promotes cell death in human versus mosquito cells. Cell Metab..

[5] Thai M (2014). Adenovirus E4ORF1-induced MYC activation promotes host cell anabolic glucose metabolism and virus replication. Cell Metab..

[6] Ayres JS (2020). A metabolic handbook for the COVID-19 pandemic. Nat. Metab..

[7] Martine-Reyes I, Chandel NS (2020). Mitochondrial TCA cycle metabolites control physiology and disease. Nat. Commun..

[8] Morita M (2013). mTORC1 controls mitochondrial activity and biogenesis through 4E-BP-dependent translational regulation. Cell Metab..

[9] Mullen AR (2012). Reductive carboxylation supports growth in tumour cells with defective mitochondria. Nature.

[10] Mullen AR (2014). Oxidation of alpha-ketoglutarate is required for reductive carboxylation in cancer cells with mitochondrial defects. Cell Rep..

[11] Sharma A (2020). Human iPSC-derived cardiomyocytes are susceptible to SARS-CoV-2 infection. Cell Rep. Med..

[12] Cheng T (2011). Pyruvate carboxylase is required for glutamine-independent growth of tumor cells. Proc. Natl Acad. Sci. USA.

[13] Purkayastha A (2020). Direct exposure to SARS-CoV-2 and cigarette smoke increases infection severity and alters the stem cell-derived airway repair response. Cell Stem Cell.

[14] Liu GY, Sabatini DM (2020). mTOR at the nexus of nutrition, growth, ageing and disease. Nat. Rev. Mol. Cell Biol..

[15] O’Shea C (2005). Adenoviral proteins mimic nutrient/growth signals to activate the mTOR pathway for viral replication. EMBO J..

[16] Meade N, King M, Munger J, Walsh D (2019). mTOR dysregulation by vaccinia virus F17 controls multiple processes with varying roles in infection. J. Virol..

[17] Klann K (2020). Growth factor receptor signaling inhibition prevents SARS-CoV-2 replication. Mol. Cell.

[18] Gordon DE (2020). A SARS-CoV-2 protein interaction map reveals targets for drug repurposing. Nature.

[19] Merad M, Martin JC (2020). Pathological inflammation in patients with COVID-19: a key role for monocytes and macrophages. Nat. Rev. Immunol..

[20] Birsoy K (2015). An essential role of the mitochondrial electron transport chain in cell proliferation is to enable aspartate synthesis. Cell.

[21] Sullivan LB (2015). Supporting aspartate biosynthesis is an essential function of respiration in proliferating cells. Cell.

[22] Krall, A. S. et al. Asparagine couples mitochondrial respiration to ATF4 activity and tumour growth. *Cell Metab.*10.1016/j.cmet.2021.02.001 (2021). 10.1016/j.cmet.2021.02.001 PMC8102379 33609439

[23] Kindrachuk J (2015). Antiviral potential of ERK/MAPK and PI3K/AKT/mTOR signaling modulation for Middle East respiratory syndrome coronavirus infection as identified by temporal kinome analysis. Antimicrob. Agents Chemother..

[24] Wang LW (2019). Epstein-Barr virus subverts mevalonate and fatty acid pathways to promote infected B-cell proliferation and survival. PLOS Pathog..

[25] Tay MZ, Poh CM, Rénia L, MacAry PA, Ng LFP (2020). The trinity of COVID-19: immunity, inflammation and intervention. Nat. Rev. Immunol..

[26] Paul MK (2014). Dynamic changes in intracellular ROS levels regulate airway basal stem cell homeostasis through Nrf2-dependent notch signaling. Cell Stem Cell.

[27] Chambers MC (2012). A cross-platform toolkit for mass spectrometry and proteomics. Nat. Biotechnol..

[28] Pluskal T, Castillo S, Villar-Briones A, Oresic M (2010). MZmine 2: modular framework for processing, visualizing, and analyzing mass spectrometry-based molecular profile data. BMC Bioinform..

[29] Myers OD, Sumner SJ, Li S, Barnes S, Du X (2017). One step forward for reducing false positive and false negative compound identifications from mass spectrometry metabolomics data: new algorithms for constructing extracted ion chromatograms and detecting chromatographic peaks. Anal. Chem..

[30] Su X, Lu W, Rabinowitz JD (2017). Metabolite spectral accuracy on orbitraps. Anal. Chem..

