# Supplementary material for: SARS-CoV-2 infection rewires host cell metabolism and is potentially susceptible to mTORC1 inhibition
Source: Nat Commun. 2021 Mar 25;12:1876. doi: 10.1038/s41467-021-22166-4 (PMC7994801; doi:10.1038/s41467-021-22166-4)
Supplement: Supplementary file 1 — Supplementary Information [file 41467_2021_22166_MOESM1_ESM.pdf]

Supplementary Information

SARS-CoV-2 infection rewires host cell metabolism and is potentially susceptible to mTORC1 inhibition

Peter J. Mullen … Vaithilingaraja Arumugaswami*, Heather R. Christofk*

*Co-corresponding authors: hchristofk@mednet.ucla.edu and Varumugaswami@mednet.ucla.edu

Supplementary Figure 1: SARS-CoV-2 infection alters glutamine entry into the TCA cycle

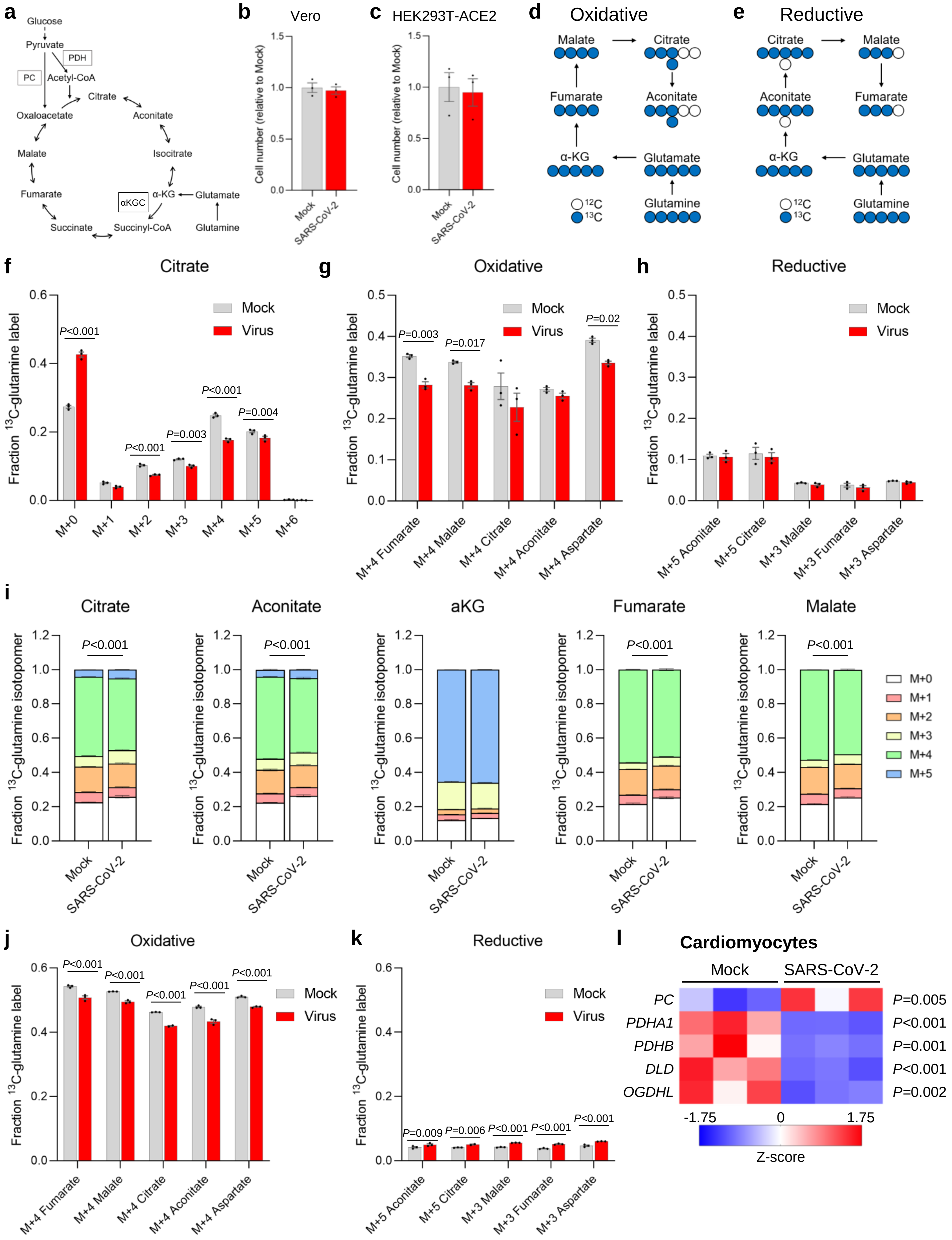

Supplementary Figure 1: SARS-CoV-2 infection alters glutamine entry into the TCA cycle. **a,** Schematic of the TCA cycle showing entry of glucose and glutamine derived carbons. PC, pyruvate carboxylase, PDH, pyruvate dehydrogenase and αKG dehydrogenase complex, αKGC, are highlighted. **b,** SARS-CoV-2 does not reduce Vero cell number after 24 hr infection. Vero cells were infected with SARS-CoV-2 (red bars) for 24 hr and then cell numbers were counted. Data are normalized to mock infected (grey bars) cells (n = 3 biologically independent samples). **c,** SARS-CoV-2 does not reduce HEK293T-ACE2 cell number after 24 hr infection. HEK293T-ACE2 cells were infected with SARS-CoV-2 (red bars) for 24 hr and then cell numbers were counted. Data are normalized to mock infected (grey bars) cells (n = 3 biologically independent samples). **d,** Schematic of oxidative U-^{13}C-glutamine carbon incorporation into the TCA cycle. ^{12}Carbons are shown as white circles and ^{13}Carbons are shown as blue circles. **e,** Schematic of reductive glutamine U-^{13}C-glutamine carbon incorporation into the TCA cycle. ^{12}Carbons are shown as white circles and ^{13}Carbons are shown as blue circles. **f,** U-^{13}C-glutamine labeling of citrate is decreased in SARS-CoV-2 infected cells. Oxidative M+4 citrate is decreased and reductive M+5 citrate is maintained in SARS-CoV-2 infected (red bars) Vero cells compared with mock infected (grey bars) Vero cells after 24 hr incubation with ^{13}C-glutamine (n = 3 biologically independent samples). **g,** The oxidative TCA cycle is decreased in SARS-CoV-2 infected (red bars) Vero cells compared with mock infected (grey bars) Vero cells after 14 hr infection (n = 3 biologically independent samples). **h,** The reductive TCA cycle is maintained in SARS-CoV-2 infected (red bars) Vero cells compared with mock infected (grey bars) Vero cells after 14 hr infection (n = 3 biologically independent samples). **i,** Glutamine entry into the TCA cycle is reduced in SARS-CoV-2 infected HEK29T-ACE2 cells. Mass isotopomer analysis of TCA cycle metabolites in mock or SARS-CoV-2 infected HEK293T-ACE2 cells after 24 hr incubation with U-13C-glutamine. Each isotopomer is a different colour, which is defined in the figure. *P* values compare the total fraction labeled between mock and SARS-CoV-2 infected cells (n = 3 biologically independent samples). **j,** The oxidative TCA cycle is decreased in SARS-CoV-2 infected (red bars) HEK293T-ACE2 cells compared with mock infected (grey bars) HEK293T-ACE2 cells after 24 hr infection (n = 3 biologically independent samples). **k,** The reductive TCA cycle is maintained in SARS-CoV-2 infected (red bars) HEK293T-ACE2 cells compared with mock infected (grey bars) HEK293T-ACE2 cells after 24 hr infection (n = 3 biologically independent samples). **l,** mRNA levels of αKGC and PDH complex members is reduced in SARS-CoV-2 infected cardiomyocytes. The statistical significance of these differentially expressed genes was measured with a false discovery rate (FDR) p-value <0.01. Data is taken from RNA-seq analysis of SARS-CoV-2 infected cardiomyocytes[11]. Unless indicated, data are the mean ± s.e.m. and *P* values were obtained by two-way ANOVA with Sidak’s multiple comparison test.

Supplementary Figure 2: SARS-CoV-2 infection alters glucose entry into the TCA cycle

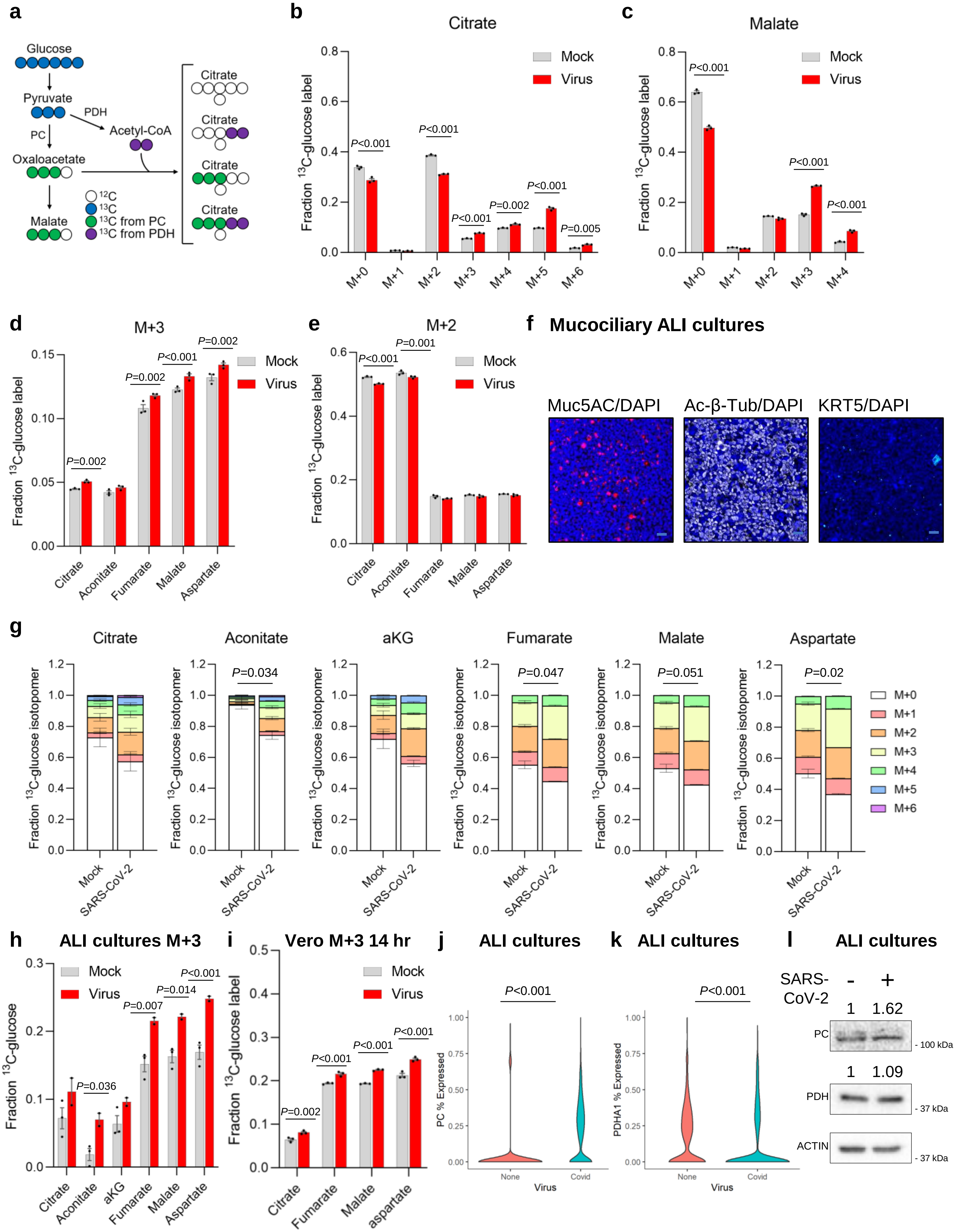

Supplementary Figure 2: SARS-CoV-2 infection alters glucose entry into the TCA cycle. **a,** Schematic of U-^{13}C-glucose carbon incorporation into the TCA cycle using PC (green circles) or PDH (purple circles). ^{12}Carbons are shown as white circles and glucose and pyruvate ^{13}Carbons are shown as blue circles **b,** Pyruvate entry to the TCA cycle using PC is increased in SARS-CoV-2 infected (red bars) Vero cells compared with mock infected (grey bars) Vero cells. M+3 and M+5 isotopomers of citrate are increased in SARS-CoV-2 infected Vero cells after 24 hr incubation with U-^{13}C-glucose (n = 3 biologically independent samples). **c,** Pyruvate entry to the TCA cycle using PC is increased in SARS-CoV-2 infected (red bars) Vero cells compared with mock infected (grey bars) Vero cells. M+3 isotopomer of malate is increased in SARS-CoV-2 infected Vero cells after 24 hr incubation with U-1^{13}C-glucose (n = 3 biologically independent samples). **d,** Pyruvate entry to the TCA cycle using PC is increased in SARS-CoV-2 (red bars) infected HEK293T-ACE2 cells compared with mock infected (grey bars) HEK293T-ACE2 cells. M+3 isotopomers are increased in SARS-CoV-2 infected HEK293T-ACE2 cells after 24 hr incubation with U-13C-glucose (n = 3 biologically independent samples). **e,** Pyruvate entry to the TCA cycle using PDH is maintained in SARS-CoV-2 infected (red bars) HEK293T-ACE2 cells compared with mock infected (grey bars) HEK293T-ACE2 cells. Citrate and aconitate M+2 isotopomers are slightly reduced in SARS-CoV-2 infected HEK293T-ACE2 cells after 24 hr incubation with U-13C-glucose (n = 3 biologically independent samples). **f,** NHBE ALI cultures are differentiated and contain mucus cells (Muc5AC expression, red), ciliated cells (Ac-β-tubulin expression, white) and airway basal stem cells (keratin 5 expression, green). Nuclei are stained with DAPI (blue). **g,** Glucose-derived carbon entry into the TCA cycle is increased in SARS-CoV-2 infected NHBE ALI cultures. Mass isotopomer analysis of TCA cycle metabolites in mock or SARS-CoV-2 infected NHBE ALI cultures after 24 hr incubation with U-^{13}C-glucose. Each isotopomer is a different colour, which is defined in the figure. *P* values compare the total fraction labeled between mock and SARS-CoV-2 infected cells (n = 3 biologically independent samples). **h,** Pyruvate entry to the TCA cycle using PC is increased in SARS-CoV-2 infected HNHBE ALI cultures. M+3 isotopomers are increased in SARS-CoV-2 infected (red bars) NHBE ALI cultures compared with mock infected (grey bars) NHBE ALI cultures after 24 hr incubation with U-^{13}C-glucose (n = 3 biologically independent samples). **i,** Pyruvate entry to the TCA cycle using PC is increased in SARS-CoV-2 infected (red bars) Vero cells compared with mock infected (grey bars) Vero cells after 14 hr infection. M+3 isotopomers are increased in SARS-CoV-2 infected Vero cells after 14 hr incubation with U-^{13}C-glucose (n = 3 biologically independent samples). **j,** PC expression is increased in SARS-CoV-2 infected ALI cultures. Data is taken from scRNA-seq analysis of SARS-CoV-2 infected ALI cultures[13]. **k,** PDHA expression is decreased in SARS-CoV-2 infected ALI cultures. Data is taken from scRNA-seq analysis of SARS-CoV-2 infected ALI cultures[13]. **l,** PC is increased at the protein level in SARS-CoV-2 infected NHBE ALI cultures. PDH is not altered at the protein level. Unless indicated, data are the mean ± s.e.m. and *P* values were obtained by two-way ANOVA with Sidak’s multiple comparison test. Scale bar 30 µM.

Supplementary Figure 3: SARS-CoV-2 infection increases mTORC1 activity

a **Vero cells**

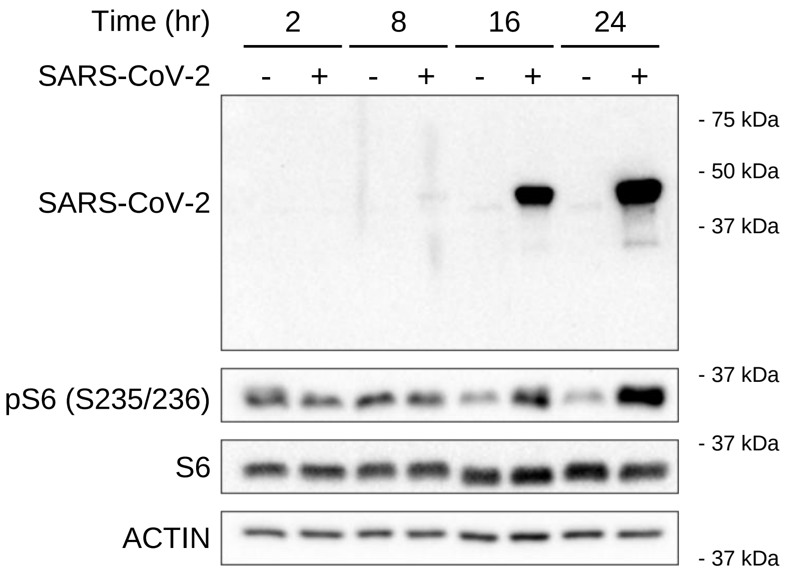

b **Mucociliary ALI cultures**

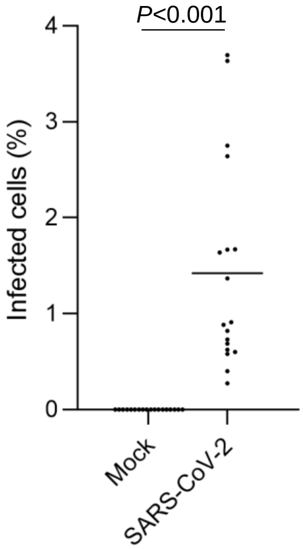

c **Vero cells (24 hr)**

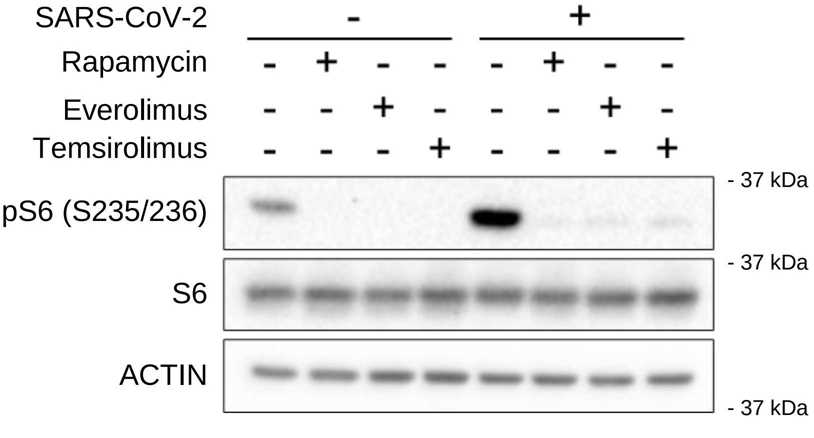

Supplementary Figure 3: SARS-CoV-2 infection increases mTORC1 activity.
a, mTORC1 activity is increased in SARS-CoV-2 infected Vero kidney epithelial cells. Protein was extracted from Vero cells infected with SARS-CoV-2 for the indicated timepoints and immunoblotted for the indicated proteins (representative of 2 biological independent samples). **b,** ALI cultures show low infectivity by SARS-CoV-2, as seen in the lungs of human patients. ALI cultures were infected with SARS-CoV-2 for 72 hr, and the cultures were stained with an anti-SARS coronavirus antibody to show SARS-CoV-2 levels and DAPI to stain the nuclei of the mucociliary cells. Percent positivity was calculated by dividing the number of SARS-CoV-2 positive cells by the total number of cells (graph shows mean, n = 5 biological replicates, each with 3–5 technical replicates). *P* value was obtained using an two-sided unpaired t test. **c,** mTORC1 inhibitors prevent mTORC1 activation in SARS-CoV-2 infected Vero cells. Cells were infected with SARS-CoV-2 and treated with 1 µM of the indicated mTORC1 inhibitors. Protein was extracted after 24 hr and immunoblotted for the indicated proteins (representative of 2 biological independent samples).

Supplementary Figure 4: mTORC1 inhibitors reduce SARS-CoV-2 replication

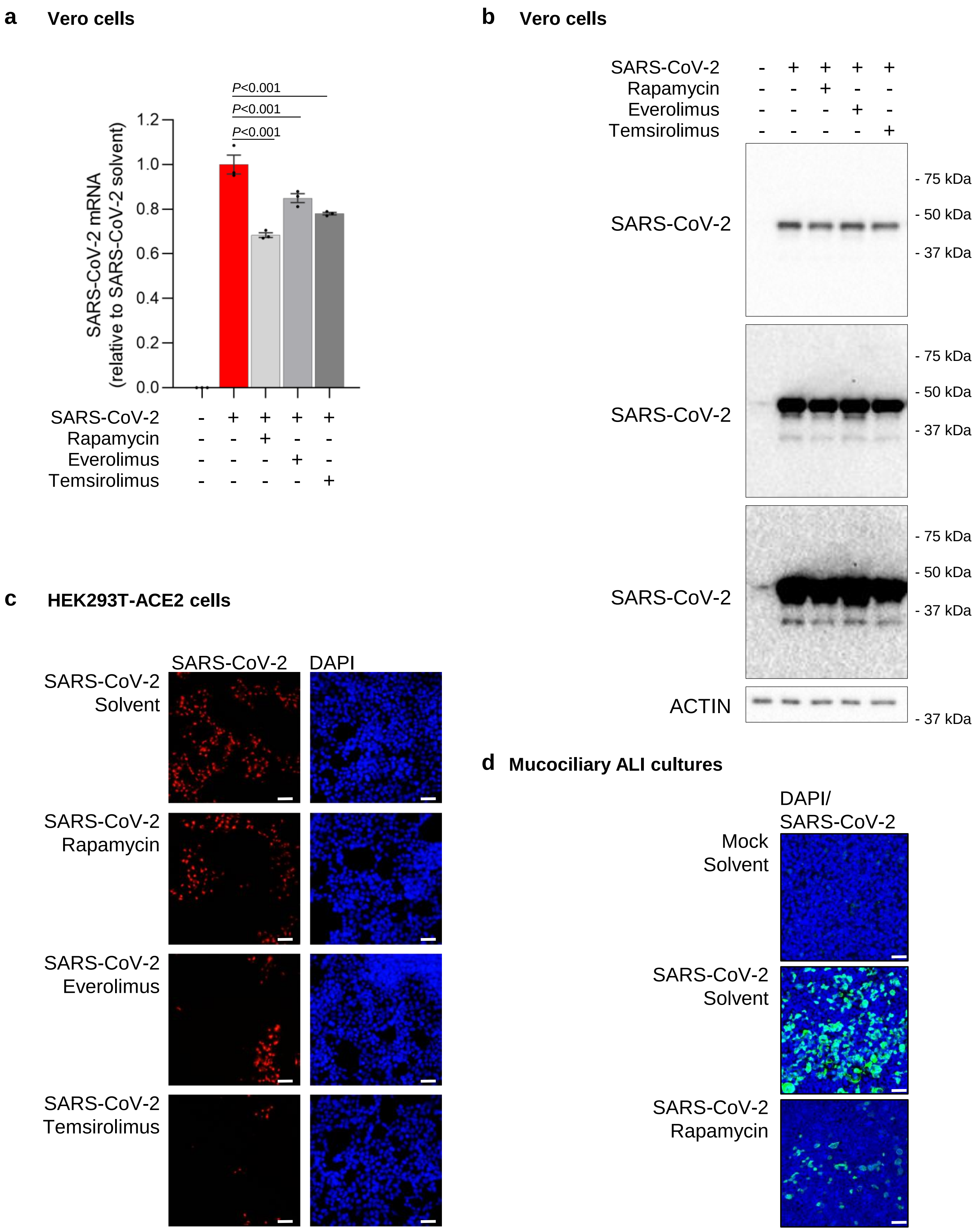

Supplementary Figure 4: mTORC1 inhibitors reduce SARS-CoV-2 replication.
a, FDA-approved mTORC1 inhibitors reduce SARS-CoV-2 gene expression. Vero cells were infected with SARS-CoV-2 (red bar) and treated with 1 µM of the indicated mTORC1 inhibitors (grey bars). RNA was extracted after 24 hr and SARS-CoV-2 N gene expression quantified relative to RPLP0 using qPCR. Data are normalized to SARS-CoV-2 solvent infected cells (n = 3 biologically independent samples. Data are mean ± s.e.m. and *P* values were obtained by one-way ANOVA with Tukey's multiple comparison's test). **b,** FDA-approved mTORC1 inhibitors reduce SARS-CoV-2 protein expression. Vero kidney epithelial cells were infected with SARS-CoV-2 and treated with 1 µM of the indicated mTORC1 inhibitors. Protein was extracted after 24 hr and immunoblotted with an anti-SARS coronavirus antibody to determine SARS-CoV-2 protein expression. Multiple exposures of SARS-CoV-2 antibody are shown (representative of 2 biological independent samples). **c,** FDA-approved mTORC1 inhibitors reduce SARS-CoV-2 levels in HEK293T-ACE2 cells. Immunofluorescence of cells infected with SARS-CoV-2 and treated with 5 µM of the indicated FDA approved mTORC1 inhibitors for 72 hr. Cells were stained for SARS-CoV S protein (red) to show SARS-CoV-2 levels. DAPI (blue) stained the nuclei of the HEK293T-ACE2 cells (n = 3 biologically independent samples). **d,** The FDA-approved mTORC1 inhibitor rapamycin reduces SARS-CoV-2 levels in NHBE ALI mucociliary cultures when added after infection. Immunofluorescence of cells infected with SARS-CoV-2 and treated with 1 µM rapamycin for 48 hr. Rapamycin was added 2 hr post infection. Cells were stained with an anti-SARS coronavirus antibody (green) to show SARS-CoV-2 levels. DAPI (blue) stained the nuclei of the mucociliary cells (n = 3 biologically independent samples). Scale bar 30 µM.

Supplementary Table 1: List of antibodies used

Antibody	Source	Information
phospho-S235/236 S6	Cell Signaling Technology	Cat#4858, immunoblot 1:3000
S6	Cell Signaling Technology	Cat#2217, immunoblot 1:1000
4EBP1	Cell Signaling Technology	Cat#9644, immunoblot 1:500
phospho-Thr37/46 4EBP1	Cell Signaling Technology	Cat#2855, immunoblot 1:500
AKT	Cell Signaling Technology	Cat#4691, immunoblot 1:1000
phospho-Ser473 AKT	Cell Signaling Technology	Cat#9271, immunoblot 1:1000
ERK	Cell Signaling Technology	Cat#9102, immunoblot 1:1000
phospho- Thr202/Tyr204 ERK	Cell Signaling Technology	Cat#4370, immunoblot 1:1000
OGDH	Cell Signaling Technology	Cat#26865, immunoblot 1:1000
DLAT	Cell Signaling Technology	Cat#12362, immunoblot 1:1000
PDH	Cell Signaling Technology	Cat#3205, immunoblot 1:1000
β-ACTIN	Cell Signaling Technology	Cat#3700, immunoblot 1:1000
DLD	Thermo Fisher	Cat# PA5-70397, immunoblot 1:1000
PC	Protein Tech	Cat#16588-1-AP, immunoblot 1:1000
SARS-CoV	BEI Resources, NIAID, NIH	Cat#NR-10361, immunoblot 1:10,000
SARS-CoV	BEI Resources, NIAID, NIH	Cat#NR-10361, immunocytochemistry 1:400
SARS-CoV S protein	BEI Resources, NIAID, NIH	Cat#NR-616, immunocytochemistry 1:100
dsRNA	Absolute Antibody	Cat#Ab01299-2.0, immunocytochemistry 1:100
phospho-Thr37/46 4EBP1	Cell Signaling Technology	Cat#2855, immunohistochemistry 1:800
CD68	Cell Signaling Technology	Cat#76437, immunohistochemistry 1:500

Supplementary Table 2 List of primers used

**Gene**	**Direction**	**Sequence**
RPLP0	Forward (5’ - 3’)	TCTACAACCCTGAAGTGCTTGAT
RPLP0	Reverse (5’ - 3’)	CAATCTGCAGACAGACACTGG
SARS-CoV-2 *N* gene	Forward (5’ - 3’)	GACCCCAAAATCAGCGAAAT
SARS-CoV-2 *N* gene	Reverse (5’ - 3’)	TCTGGTTACTGCCAGTTGAATCTG
