# Supplementary material for: SARS-CoV-2 infection rewires host cell metabolism and is potentially susceptible to mTORC1 inhibition
Source: Nat Commun. 2021 Mar 25;12:1876. doi: 10.1038/s41467-021-22166-4 (PMC7994801; doi:10.1038/s41467-021-22166-4)
Supplement: Supplementary file 2 — Reporting Summary [file 41467_2021_22166_MOESM2_ESM.pdf]

nature research

Corresponding author(s): Vaithilingaraja Arumugaswami and Heather R. Christofk

Last updated by author(s): Feb 24, 2021

Reporting Summary

Nature Research wishes to improve the reproducibility of the work that we publish. This form provides structure for consistency and transparency in reporting. For further information on Nature Research policies, see our Editorial Policies and the Editorial Policy Checklist.

Statistics

For all statistical analyses, confirm that the following items are present in the figure legend, table legend, main text, or Methods section.

n/a	Confirmed	
☐	☒	The exact sample size (*n*) for each experimental group/condition, given as a discrete number and unit of measurement
☐	☒	A statement on whether measurements were taken from distinct samples or whether the same sample was measured repeatedly
☐	☒	The statistical test(s) used AND whether they are one- or two-sided<br>*Only common tests should be described solely by name; describe more complex techniques in the Methods section.*
☒	☐	A description of all covariates tested
☐	☒	A description of any assumptions or corrections, such as tests of normality and adjustment for multiple comparisons
☐	☒	A full description of the statistical parameters including central tendency (e.g. means) or other basic estimates (e.g. regression coefficient) AND variation (e.g. standard deviation) or associated estimates of uncertainty (e.g. confidence intervals)
☐	☒	For null hypothesis testing, the test statistic (e.g. *F*, *t*, *r*) with confidence intervals, effect sizes, degrees of freedom and *P* value noted<br>*Give P values as exact values whenever suitable.*
☒	☐	For Bayesian analysis, information on the choice of priors and Markov chain Monte Carlo settings
☒	☐	For hierarchical and complex designs, identification of the appropriate level for tests and full reporting of outcomes
☒	☐	Estimates of effect sizes (e.g. Cohen's *d*, Pearson's *r*), indicating how they were calculated

Our web collection on statistics for biologists contains articles on many of the points above.

Software and code

Policy information about availability of computer code

Data collection	No custom software was used
Data analysis	We used MZmine 2 to analyze the metabolomics data. We used FIJI to quantify immunoblots, Graphpad version 9 for graphs and statistical analysis and QuPath v0.2 to analyze immunohistochemtry samples

For manuscripts utilizing custom algorithms or software that are central to the research but not yet described in published literature, software must be made available to editors and reviewers. We strongly encourage code deposition in a community repository (e.g. GitHub). See the Nature Research guidelines for submitting code & software for further information.

Data

Policy information about availability of data

All manuscripts must include a data availability statement. This statement should provide the following information, where applicable:

- Accession codes, unique identifiers, or web links for publicly available datasets
- A list of figures that have associated raw data
- A description of any restrictions on data availability

The authors declare that the data supporting the findings of this study are available within the paper or are available from the corresponding author (H.R.C.) upon reasonable request.

Field-specific reporting

Please select the one below that is the best fit for your research. If you are not sure, read the appropriate sections before making your selection.

- [x] Life sciences
- [ ] Behavioural & social sciences
- [ ] Ecological, evolutionary & environmental sciences

For a reference copy of the document with all sections, see nature.com/documents/nr-reporting-summary-flat.pdf

Life sciences study design

All studies must disclose on these points even when the disclosure is negative.

Sample size	We did not calculate the required sample size, instead we used the standard 3 biological replicates for qPCR, IF and metabolomic experiments. These sample sizes are considered appropriate to determine biologically relevant differences with the techniques we used in this study.
Data exclusions	No data were excluded.
Replication	All experiments were replicated in independent experiments, typically 2-3 times. The number of experiments performed is indicated in the figure legends.
Randomization	We used different treatment maps for each cell culture plate and experiment. This ensured that the the position of the wells did not influence the interpretation of the data.
Blinding	The investigator measuring the metabolites was blinded to the groups.

Reporting for specific materials, systems and methods

We require information from authors about some types of materials, experimental systems and methods used in many studies. Here, indicate whether each material, system or method listed is relevant to your study. If you are not sure if a list item applies to your research, read the appropriate section before selecting a response.

Materials & experimental systems

n/a	Involved in the study	
☐	☒	Antibodies
☐	☒	Eukaryotic cell lines
☒	☐	Palaeontology and archaeology
☒	☐	Animals and other organisms
☐	☒	Human research participants
☒	☐	Clinical data
☒	☐	Dual use research of concern

Methods

n/a	Involved in the study	
☒	☐	ChIP-seq
☒	☐	Flow cytometry
☒	☐	MRI-based neuroimaging

Antibodies

Antibodies used	phospho-S235/235 S6 ribosomal protein (Cell Signaling Technology 4858, 1:3000), S6 ribosomal protein (Cell Signaling Technology 2217, 1:1000), 4E-BP1 (Cell Signaling Technology 9644, 1:500) phospho-Thr37/46 4E-BP1 (Cell Signaling Technology 2855, 1:500 for immunoblots, 1:800 for immunohistochemistry),AKT (Cell Signaling Technology 4691, 1:1000), phospho-Ser473 AKT (Cell Signaling Technology 9271, 1:1000), ERK (Cell Signaling Technology 9102, 1:1000), phospho- Thr202/Tyr204 ERK (Cell Signaling Technology 4370, 1:1000), OGDH (Cell Signaling Technology 26865, 1:1000), DLD (Thermo Fisher PA5-70397, 1:1000), DLAT (Cell Signaling Technology 12362, 1:1000), PC (Protein Tech 16588-1-AP, 1:10,000), PDH (Cell Signaling Technology 3205, 1:1000), SARS-CoV antibody (BEI Resources, NIAID, NIH, NR-10361, 1:10,000 for immunoblots, 1:400 for immunocytochemistry), anti-SARS-CoV S Protein Similar to 240C (BEI Resources, NIAID, NIH, NR-616, 1:100), anti-dsRNA (Absolute Antibody, Ab01299-2.0, 1:100), CD68 (Cell Signaling Technology 76437, 1:500) and β-ACTIN (Cell Signaling Technology 3700, 1:1000).
Validation	All Cell Signaling Technology antibodies are commonly used in studies and adhere to the Hallmarks of Antibody Validation™, six complementary strategies that can be used to determine the functionality, specificity, and sensitivity of an antibody in any given assay. CST adapted the work by Uhlen, et. al., ("A Proposal for Validation of Antibodies." Nature Methods (2016)) to build the Hallmarks of Antibody Validation, based on their decades of experience as an antibody manufacturer and our dedication to reproducible science. Thermo Fisher has collaborated with Nature to highlight Thermo Fisher's commitment to antibody validation, and involves a two step process of target specificity and functional validation. Protein Tech validates antibodies using siRNA. The Absolute Antibody antibody was originally characterized and validate in 'J Schönborn, J Oberstrass, E Breyel, J Tittgen, J Schumacher, and N Lukacs Monoclonal antibodies to double-stranded RNA as probes of RNA structure in crude nucleic acid extracts. Nucleic Acids Res. 1991 Jun 11; 19(11): 2993–3000 PMID:2057357'. BEI Resources confirmed the specificity of the SARs-CoV antibodies by ELISA and virus neutralization assays.

Eukaryotic cell lines

Policy information about cell lines

Cell line source(s)	Vero(ATCC CRL1586) and HEK293T cells (ATCC CRL3216) were bought from ATCC. NHBE cells (CC-2540) were bought from Lonza.
Authentication	All cell lines were bought directly from ATCC. ATCC uses morphology, karyotyping, and PCR based approaches to confirm the identity of human cell lines and to rule out both intra- and interspecies contamination. These include an assay to detect species specific variants of the cytochrome C oxidase I gene (COI analysis) to rule out inter-species contamination and short tandem repeat (STR) profiling to distinguish between individual human cell lines and rule out intra-species contamination. NHBE cells were validated by confirming their ability to differentiate in our ALI cultures using IF.
Mycoplasma contamination	All cell lines were routinely tested for mycoplasma and tested negative
Commonly misidentified lines (See ICLAC register)	No commonly misidentified cell lines were used in this study

Human research participants

Policy information about studies involving human research participants

Population characteristics	We did not have access to clinical information about the patients, as this was outside of our REB approval
Recruitment	Participants were selected based on access to autopsy material from COVID-19 patients. Normal samples were matched by clinicians as the authors did not have access to patient information
Ethics oversight	UCLA COVID Prioritization and Feasibility (SP&F) Committee approved our request for patient material in conjunction with Pathology and Laboratory Medicine

Note that full information on the approval of the study protocol must also be provided in the manuscript.
